# Artificial intelligence-derived coronary artery calcium scoring saves time and achieves close to radiologist-level accuracy accuracy on routine ECG-gated CT

**DOI:** 10.1007/s10554-024-03306-5

**Published:** 2024-12-16

**Authors:** Jordan H. Chamberlin, Sameer Abrol, James Munford, Jim O’Doherty, Dhiraj Baruah, U. Joseph Schoepf, Jeremy R. Burt, Ismail M. Kabakus

**Affiliations:** 1 Division of Cardiothoracic Radiology, Department of Radiology and Radiological Science, Medical University of South Carolina, Clinical Science Building, 96 Jonathan Lucas Street, Suite 210, MSC 323, Charleston, SC 29425 USA; 2 Division of Cardiothoracic Radiology, Department of Radiology and Imaging Sciences, University of Utah, Salt Lake City, UT USA; 3 Siemens Medical Solutions, Malvern, PA USA

**Keywords:** Artificial intelligence, Coronary artery disease, Cardiac computed tomography, Calcium score

## Abstract

Artificial Intelligence (AI) has been proposed to improve workflow for coronary artery calcium scoring (CACS), but simultaneous demonstration of improved efficiency, accuracy, and clinical stability have not been demonstrated. 148 sequential patients who underwent routine calcium-scoring computed tomography were retrospectively evaluated using a previously validated AI model (*syngo*. CT CaScoring VB60, Siemens Healthineers, Forscheim, Germany). CACS was performed by manual (Expert alone), semi-automatic (AI + expert review), and automatic (AI alone) methods. Time to complete and intraclass correlation coefficients were the primary endpoints. Secondary endpoints included differences in multiethnic study of atherosclerosis (MESA) percentiles and stratification by calcium severity. AI and expert CACS agreement was excellent (ICC = 0.951; 95% CI 0.933–0.964). The global median time was 15 ± 2 s for AI (“Automatic”), 38 ± 13 s for the AI + manual review (“Semiautomatic”) and 45 ± 24 s for the manual segmentation. Automatic segmentation was faster than manual segmentation for all CACS severities (*P* < 0.001). AI computational time was independent of calcium burden. Global mean bias in Agatston score across all patients was 7.4 ± 102.6. The mean bias for global MESA score percentile was 2.1% ± 12%. 95% of error corresponded to a ± 10% difference in MESA score. The use of AI for CACS performs excellent accuracy, saves approximately 60% of time in comparison to manual review, and demonstrates low bias for clinical risk profiles. Time benefits are magnified for patients with high CACS. However, a semi-automatic approach is still recommended to minimize potential errors while maintaining efficiency.

## Introduction

Coronary artery disease (CAD) has remained the leading cause of mortality in the United States for decades. Coronary calcium scoring (CACS) is a commonly used non-invasive method of evaluating the presence and extent of CAD [1]. A quantitative CACS, or Agatston score, is generated using attenuation data found within cardiac computed tomography (CT) examinations and can be used for risk stratification. Larger Agatston scores, alone and as part of the Multiethnic Study of Atherosclerosis (MESA) risk score, have been shown to correlate with an increased risk of experiencing morbidity or mortality [2, 3]. Indeed, inclusion of calcium score into clinical risk scores improves risk prediction, providing rationale for CACS as a routine component of cardiac CT examinations [4, 5]. MESA score estimates a patient’s 10-year risk of coronary heart disease by integrating coronary artery calcium from the Agatston score with other clinical risk factors such as age, gender, cholesterol levels, and more. This approach provides a more comprehensive risk prediction compared to using the Agatston score alone, which quantifies calcified plaque burden [5].

In the context of CT, the definition of coronary calcium is strictly defined as coronary artery voxels measuring ≥ 130 Hounsfield units, with Agatston scores including density as a second transformation [6]. In its current clinical application, cardiac imagers must manually evaluate each coronary lesion on a slice-by-slice basis – a time consuming task, while also reporting accurate absolute values and patient-specific risk information [7]. Therefore, calcium scoring is an excellent candidate for the implementation of automated AI applications.

Multiple models exist for automated CACS that have been validated for accuracy [8]. Some examples of these methods include rule-based models, machine learning, and deep learning models. One review references at least nine studies that have investigated different methods of automated CACS with accuracies ranging from 79 to 99.98% with semi-automated approaches [9]. While these studies have demonstrated favorable performance for automated CACS, studies simultaneously investigating the accuracy and time efficiency are limited. A relatable challenge for the cardiac imager is segmenting examinations on patients with large calcium burdens and multiple plaques, where evaluation of each lesion is needed to ensure the calcium burden is in the coronary lumen and not a common error such as calcified aortic root, mitral annulus, or calcific fat [9]. Furthermore, studies evaluating the clinical impact of error are lacking and are essential before routine clinical use.

Therefore, this study aims to evaluate the performance and efficiency of a previously externally validated AI model for automatic quantification of CACS while also evaluating the impact on resulting risk scores [10]. The authors hypothesize that the AI model will assist diagnosticians with time efficiency while maintaining a high degree of accuracy and with minimal clinical reclassification.

## Materials and methods

### Ethical approval

This study was approved by the Institutional Review Board at the performing institution and the need for written informed consent was waived (Pro00133775). Patient data, including identifiers, were stored in compliance with the Health Insurance Portability and Accountability Act in an encrypted web database in institutional storage. Patient data were de-identified prior to statistical analysis, and no individual patient information was shared with third-party entities.

### Patient population

150 sequential patients from September – November 2023 who underwent routine electrocardiogram (ECG) gated non-contrast CT for the purpose of calcium scoring and risk stratification were retrospectively evaluated. Eligibility criteria included patients ≥ 18 years of age with adequate demographic variables for risk score analysis available in the electronic medical record. Patients with coronary artery stents, prior coronary artery bypass, implanted permanent pacemakers/cardioverter-defibrillator devices, and valve replacements were excluded. Two patients lacked appropriate demographics and therefore 148 patients were included in the final analysis.

### Calcium score CT acquisition

To acquire CACS, we utilized a standardized ECG-gated CT protocol. All scans were performed using multi-detector CT scanners. The scanner settings were adjusted to achieve the optimal balance between image quality and radiation dose, using a tube voltage of 120 kVp, auto mA with dose modulation, a slice thickness of 3 mm, a reconstruction interval of 1.5 mm, an adjusted field of view, and a gantry rotation time of 0.25 s.

Prospective ECG-gating was employed to minimize motion artifacts by synchronizing image acquisition with the diastolic phases of the cardiac cycle. ECG data were continuously monitored, and images were acquired during mid-diastole. Patients were instructed to hold their breath during image acquisition to reduce motion artifacts.

The raw data were reconstructed into axial images with a 512 × 512 matrix and transferred to a dedicated workstation equipped with calcium scoring software (Syngo.via, Siemens Healthineers, Malvern, PA). Coronary artery calcium was identified and quantified using the Agatston method, with voxels measuring ≥ 130 Hounsfield units being classified as calcified [11]. The Agatston score was calculated by summing the scores from all identified calcified lesions.

### Manual calcium score evaluation

Axial calcium score series were exported to a commercially available cloud-based platform for standard analysis as used in the institutional clinical practice (*syngo*. CT CaScoring VB60, Siemens Healthineers, Forscheim, Germany). A stopwatch or equivalent electronic device was used to record time taken to manually segment coronary artery calcium beginning with study launch. Two readers independently performed the manual scoring without repetition (JHD, radiology resident with 5 years of advanced cardiovascular imaging research experience and IMK, a fellowship-trained radiologist with over 10 years of experience). Upon completion of the calcium segmentation to the satisfaction of the reader both the time and calcium score were recorded.

### Automatic calcium score evaluation

Axial calcium score series were exported to the same commercially available cloud-based platform and evaluated using the calcium scoring AI module (syngo. CT CaScoring VB60, Siemens Healthineers, Forscheim, Germany). Again, a stopwatch was used to record time taken to automatically segment coronary artery calcium beginning with study launch. Time was concluded when the algorithm finished calculation of calcium score as monitored by the progress bar and ultimate availability of results. Both time and AI-derived calcium score were recorded.

### Semi-automatic calcium score evaluation

Following automatic calcium scoring and pause of the timer for recording of results, the timer was restarted and manually reviewed by one of two readers for accuracy and manual correction. Upon satisfactory review and correction of the AI-based quantification the timer was again stopped and the time recorded.

### Deep learning model

An in-depth description of the deep learning model has been previously presented and externally validated in a multi-institutional dataset [12, 13]. Briefly, the calcium scoring algorithm utilized a two-step approach: identification of calcified voxels predicted as belonging to coronary arteries by 130 Hounsfield units intensity thresholding and coronary artery territory mapping using multilayered neural networks. This algorithm was trained on 1261 routine calcium scoring scans from clinical sites across USA, Europe, and Asia and subsequently validated on 500 patients for accuracy and time versus semiautomated scoring [13], and 1171 patients for multi-site robustness and vessel-specific accuracy [12].

### Statistical analysis

A pre-test power calculation was performed based off observed AI results from previous work in Yacoub et al. [14] and Chamberlin et al. [10]. Regarding agreement, based off the previous intraclass correlation coefficient (ICC) for expert and AI performance of 0.9, a total sample size of 77 patients was required for repeatability within 10% of the previous ICC (0.8-1.0). Regarding time, assuming a 22% reduction in time with a standard deviation of 50% of the required time and at least a moderate effect size (0.3), at least 131 patients were required to detect a statistically significant change in time.

Quantitative agreement analysis for Agatston score was performed using Two-way ICCs with mixed effects and absolute agreement. Qualitative agreement for Agatson score severity was performed using Gwet’s agreement coefficient. Gwet’s AC was chosen as score severity is an ordinal variable for which magnitude of misclassification is an additional consideration versus Cohen’s Kappa and the distribution of ordinal severity data was unevenly distributed. Comparison of time per method was performed using pairwise Wilcoxon signed rank tests. Bland Altman statistics were calculated across each severity level and for MESA scores. Subsequent analysis of outliers for Agatston scores was performed. All statistics were performed in R v. 4.1.2 (R Foundation for Statistical Computing, Vienna, Austria). A p-value of less than 0.05 was considered statistically significant.

## Results

A total of 148 sequential patients were included in this study. The median age was 61 (IQR ± 14) and 79 (53.4%) were female. 136 (91.9%) of patients white, 8 (5.4%) identified as Black or African American, 2 (1.4%) identified as Asian, and a further 2 were grouped together as “other.” (Table 1).

**Table 1:** Demographics

*N* = 148	Median (IQR) | *N* (%)
Age	61 (14)
Sex	
Female Male	79 (53.4) 69 (46.6)
Race	
Asian Black White Other	2 (1.4) 8 (5.4) 136 (91.9) 2 (1.4)

**Fig. 1:**
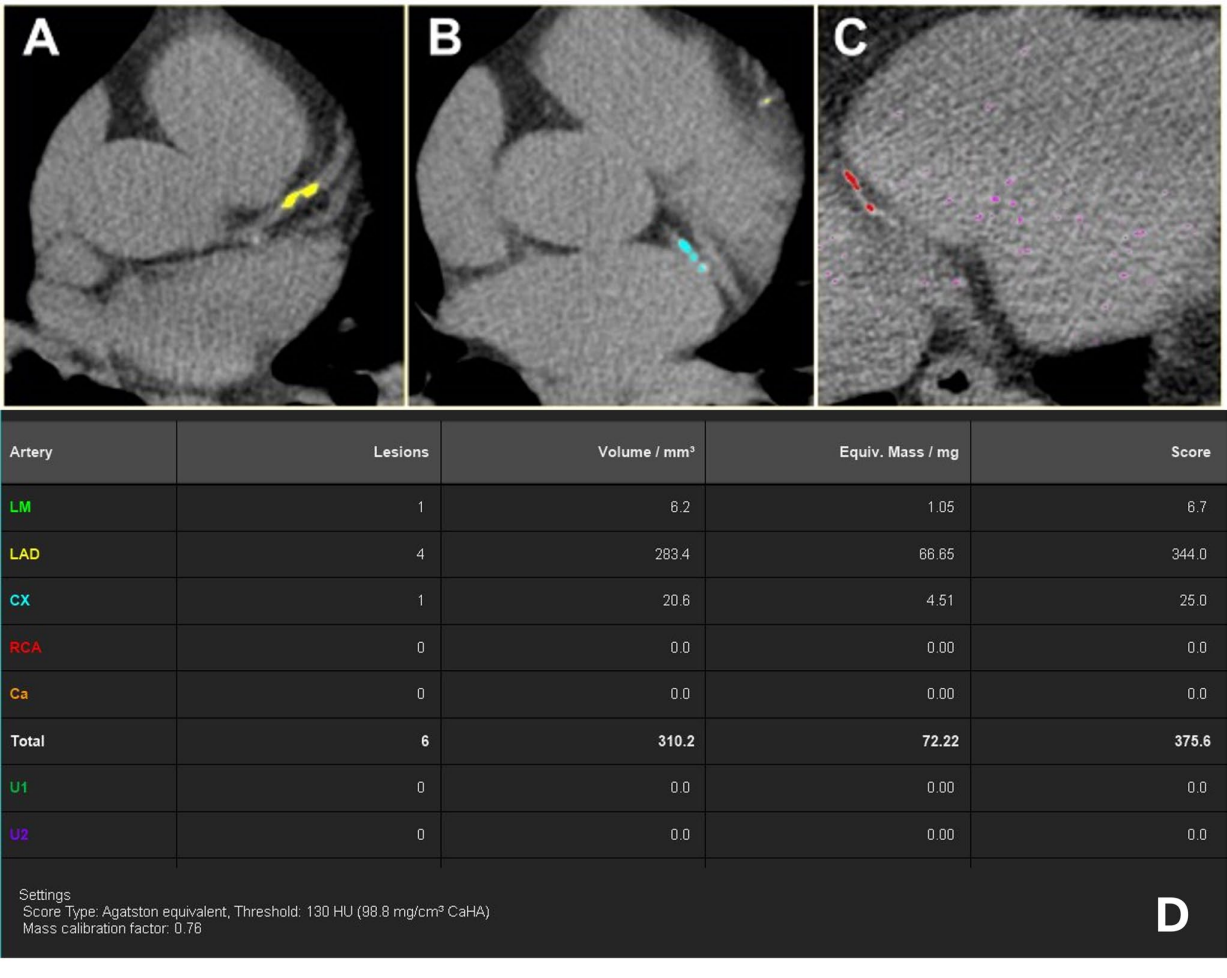
Example of AI-derived CAC quantification. **A.** True positive identification of calcific plaque in the proximal left anterior descending coronary artery (LAD). **B.** Correct identification of calcifications within the left circumflex coronary artery (CX). **C.** Correct identification of calcifications in the posterior right coronary artery (RCA). **D. ** Quantitative table demonstrating the total calcium score stratified by coronary artery territory. Both the expert and the AI agreed on an Agatston score of 547 with the same lesions. (LM = Left main coronary artery, not pictured. Ca = Calcium, U1 and U2 = User-defined ROIs, unused)

**Fig. 2:**
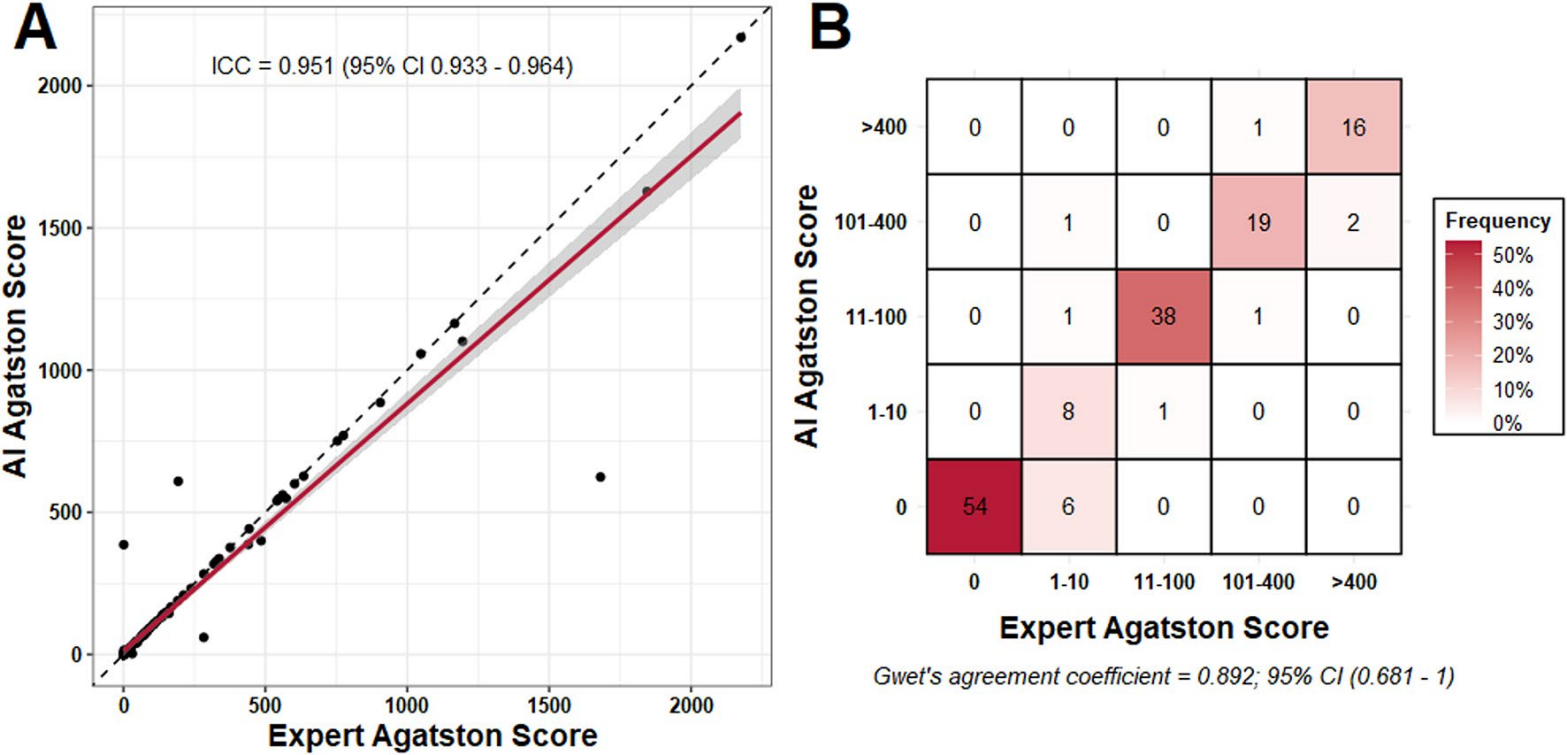
Interrater Reliability. **A.** Quantitative estimation of agreement. Two-way ICC = 0.951; 95% CI (0.933–0.964). There is a slight underestimation of the extremely large calcium scores by the AI algorithm in comparison to the expert category. (**B.**) The inter-rater reliability may be regarded as “excellent” or within “inter-expert variation”

AI and radiologist-derived Agatston scores exhibited excellent inter-rater reliability (ICC = 0.951; 95% CI 0.933–0.964). An example of AI-derived CAC quantification can be seen in Fig. 1. A slight preponderance of the AI to underestimate extremely high Agatson (> 1000) scores was observed (Fig. 2A). There was also excellent inter-rater reliability for assigning patients into an Agatston score severity classification (minimal = 1–10, mild = 10–100, etc.) as measured by Gwet’s agreement coefficient (AC1 = 0.892; 95% CI = 0.681–1) (Fig. 2B).

**Fig. 3:**
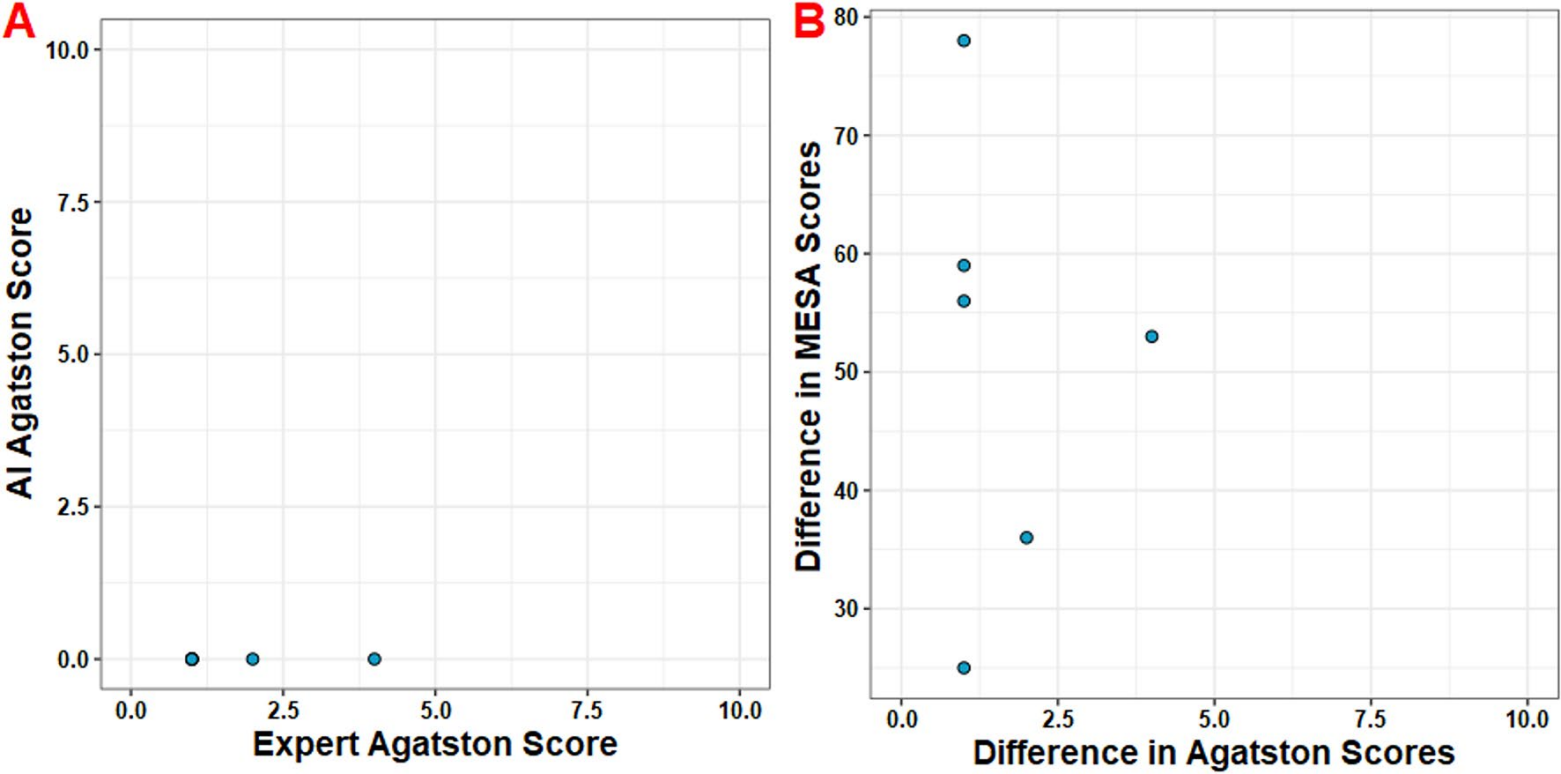
Evaluation of the previously identified 6 outliers demonstrating that small (< 5 Agatston score) differences may lead to wild swings in MESA percentile. All 6 positive outliers had an Agatston score of < 5 by expert calculation, where the AI found zero. This resulted in a 25–78% difference in MESA percentile despite the calcium discrepancy being so small

**Fig. 4:**
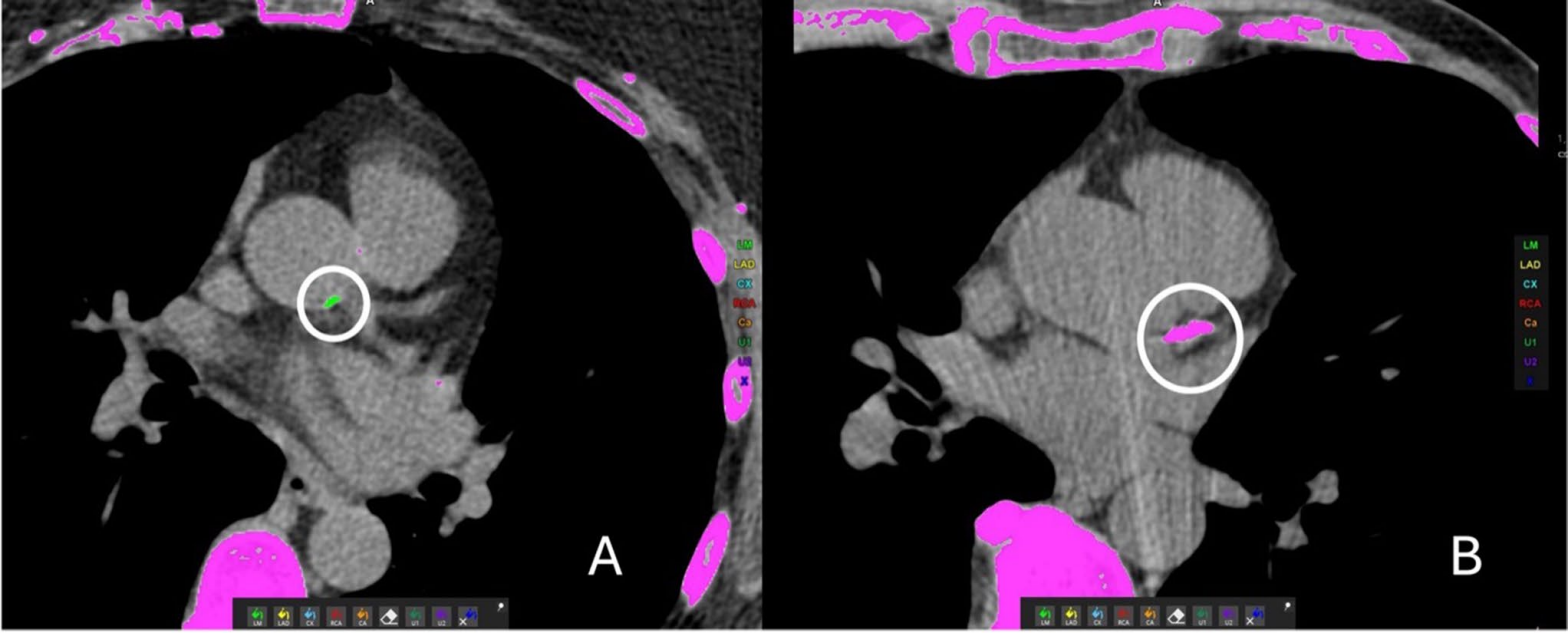
**A.** False positive detection by AI algorithm. The algorithm incorrectly identifies calcium deposits (encircled) in the sinus of Valsalva as coronary calcification. **B.** False negative. The algorithm fails to identify a calcium deposit in the left anterior descending artery (encircled)

The absolute categorical accuracy was 91.2%. By far the most frequent observation was that of a concordant zero Agatston score. 54 patients were noted by radiologists to have no coronary calcium, a finding that was perfectly replicated by the AI (54 AI/54 Radiologist; 100%). 6 patients were classified by the AI as having an Agatston score of zero when the true value was greater than zero. These 6 (4.1%) patients were all found to have Agatston scores of < 4 on root cause analysis, with discrepancies on the level of single voxels (Fig. 3A). A further 7 patients (4.8%) were discordant within one category level (E.g, mild to moderate). (Figs. 2B and 4).

**Fig. 5:**
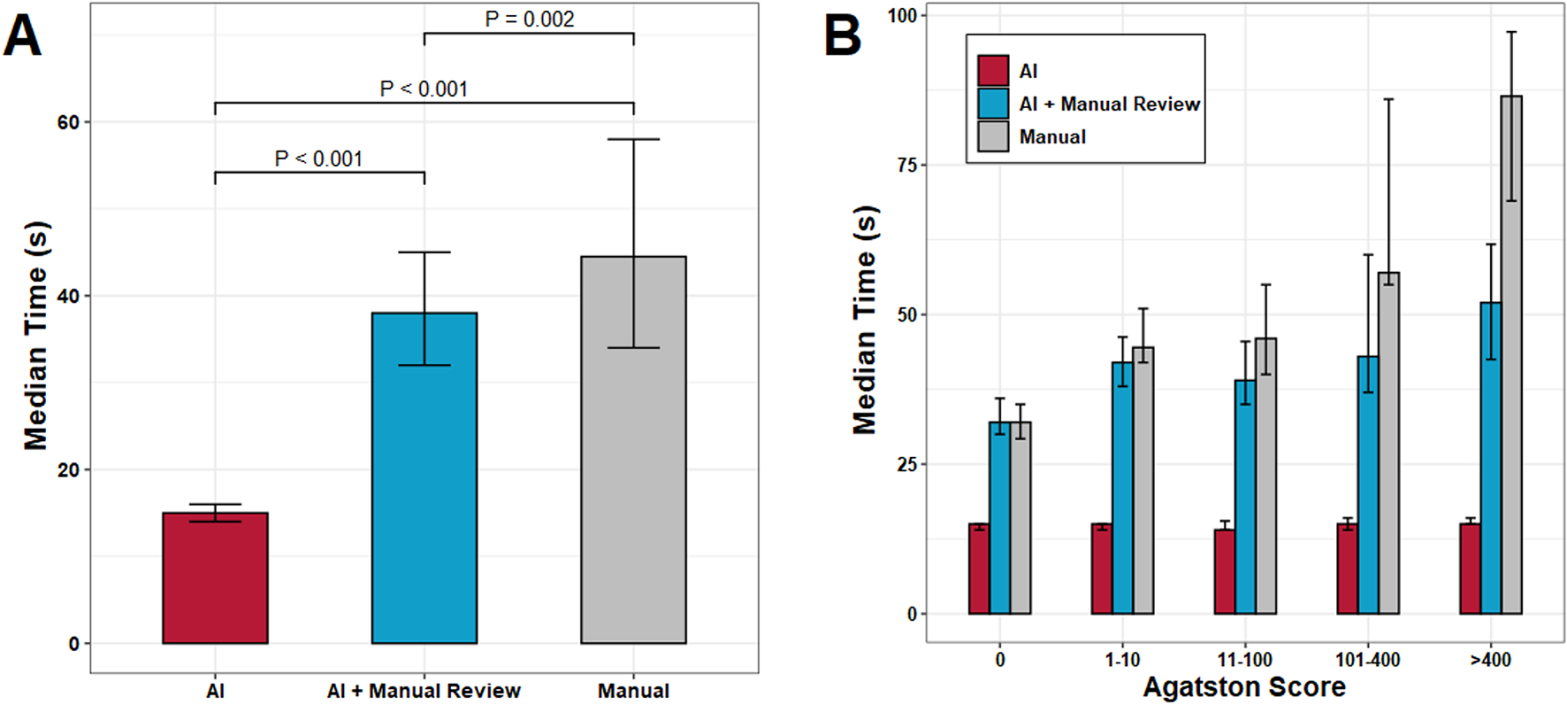
Time analysis. **A.** Comparison of all patients by median time and method. It was significantly faster for AI to measure calcium score alone than with expert alone or with AI + expert manual review (15 ± 2s vs. 38 ± 13s vs. 45 ± 24s, *p* < 0.001 for both comparisons). Additionally, AI + expert manual review was faster than expert manual segmentation alone (*p* = 0.002). **B.** When stratifying by Agatston score thresholds, AI was faster than AI + manual review or expert segmentation alone for all categories (*P* < 0.001 for all). When comparing only AI + manual review to expert segmentation alone, there was a significant reduction in time for patients with at least mild calcium burden. (p_mild_ = 0.004, p_moderate_ = 0.001, p_severe_ < 0.001)

Regarding time to perform the exam, the global median time (s) for AI (“Automatic”) was 15 ± 2, 38 ± 13 for the AI + manual review (“Semiautomatic”), and 45 ± 24 for the manual segmentation by radiologist. Time for automatic assessment by the AI algorithm was noted to be independent of Agatston score while radiologist assessment times positively correlated with Agatston score. Both automatic (*p* < 0.001) and semiautomatic (*p* = 0.002) were faster than manual assessment. Using automated assessment alone saved 29 ± 24 s (66 ± 18%) in comparison to manual assessment. (Table 2; Fig. 5A) When stratified by calcium burden, the automatic assessment was significantly faster (*p* < 0.001 for all) than manual assessment. Semiautomatic assessment was found to be significantly faster for mild (*p* = 0.004), moderate (*p* = 0.001), and severe (*p* < 0.001) Agatston scores (Table 2; Fig. 5B).

**Table 2:** Time to perform each method with stratification by calcium score. AI alone was faster for all patients. Use of a semiautomated (AI + manual review) method was faster for patients with at least a mild calcium burden. Auto: Automated method, AI alone. Semiauto: Automated method reviewed by the expert

	AI	AI + Manual Review	Manual	AI Versus Manual	
	Median ± IQR	Median ± IQR	Median ± IQR	p _Auto_	p _Semiauto_
Time (s)	15 ± 2	38 ± 13	45 ± 24	**< 0.001**	**0.002**
Time saved vs. manual (s)	29 ± 24	4 ± 15	---	---	---
Time saved vs. manual (%)	66 ± 18	10 ± 3	---	---	---
Time (Agatston = 0) (s)	15 ± 1	32 ± 6	32 ± 6	**< 0.001**	0.495
Time (Agatston = 1–10) (s)	15 ± 1	42 ± 8	45 ± 9	**< 0.001**	0.212
Time (Agatston = 11–100) (s)	14 ± 2	39 ± 11	46 ± 15	**< 0.001**	**0.004**
Time (Agatston = 101–400) (s)	15 ± 2	43 ± 23	57 ± 31	**< 0.001**	**0.001**
Time (Agatston > 400) (s)	15 ± 1	52 ± 19	87 ± 28	**< 0.001**	**< 0.001**

**Fig. 6:**
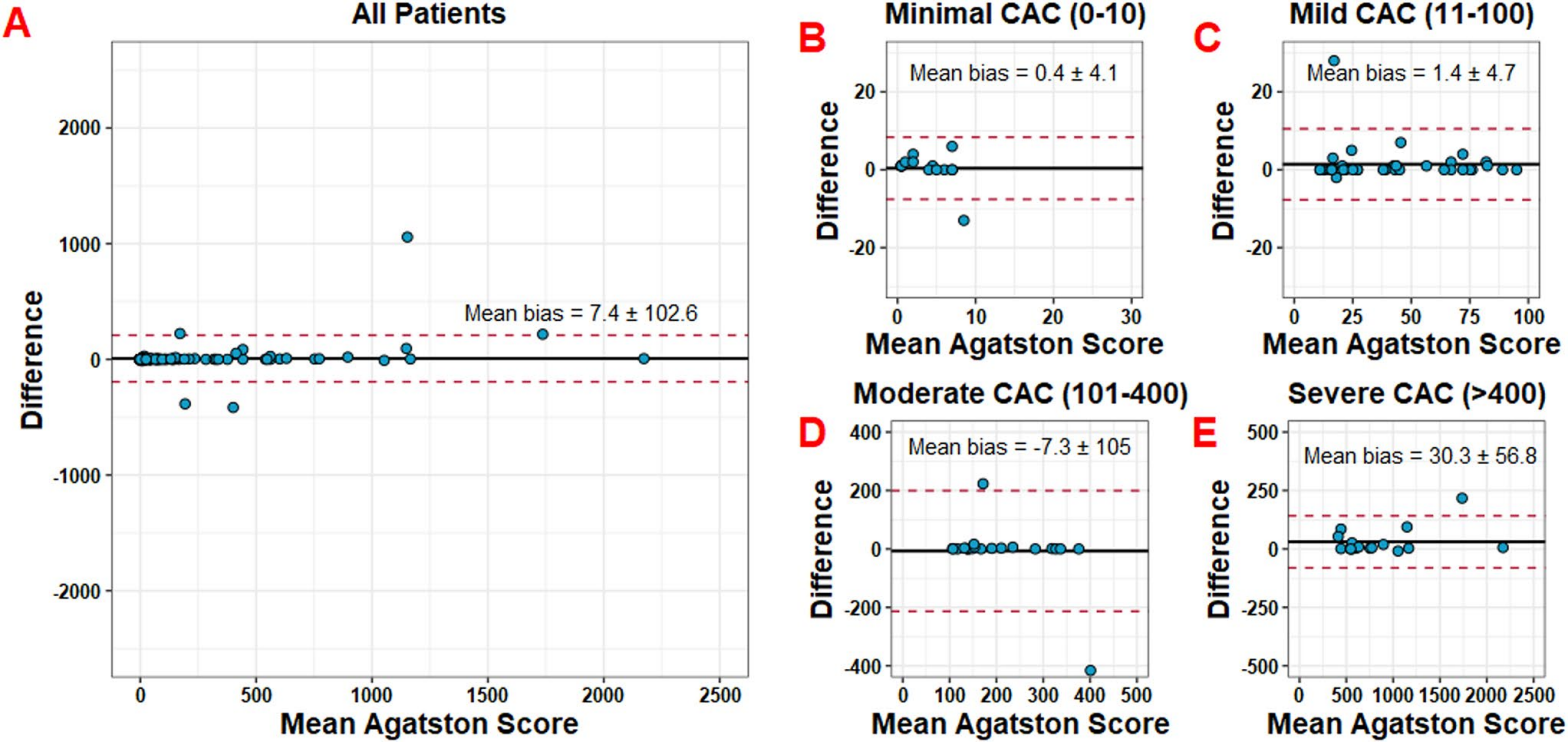
Bland-Altman statistics for Agatsston scores. **A.** All patients: mean bias = 7.4 ± 102.6. **B.** Patients with “minimal” CAC: mean bias = 0.4 ± 4.1. **C.** Patients with “mild” CAC: mean bias = 1.4 ± 4.7. **D. ** Patients with “moderate” CAC: mean bias = -7.3 ± 105. **E.** Patients with “severe” CAC: mean bias = 30.3 ± 56.8

**Fig. 7:**
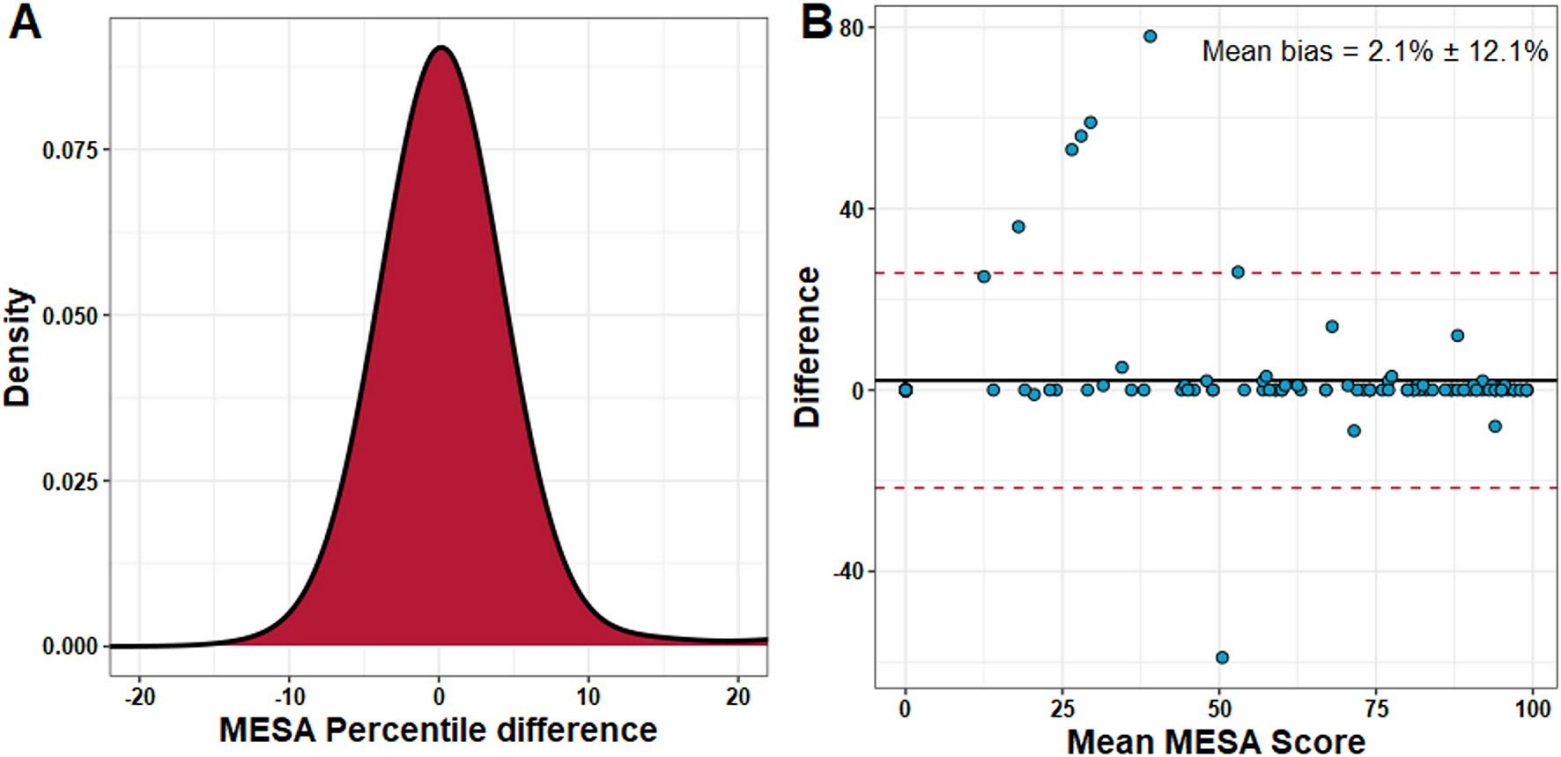
MESA Reliability. **A.** Distribution of difference in MESA percentile by AI and expert Agatston score. The distribution of error approximates normality. >2 standard deviations of error are between − 10 and 10%. **B.** Bland-Altman plot for distribution of differences. An outlier group is noted with high differences observed in MESA score. The mean bias of the whole cohort is 2.1% ± 12.1%

Bias assessment was performed with Bland-Altman statistics for both global and severity-stratified Agatston score. Global mean bias across all patients was 7.4 ± 102.6. Mean bias increased from 1.4 ± 4.7 at minimal (0–10) Agatston scores to 30.3 ± 56.8 at severe (> 400) Agatston scores (Fig. 6). Evaluation of bias of AI measurement on corresponding MESA Score was also performed. The distribution of error was found to be approximately normal with 95% of error corresponding to a ± 10% difference in MESA score percentile (Fig. 7A). The mean bias for global MESA score percentile was 2.1 ± 12%. (Fig. 7B). 6 patients were found to be outliers and correlated to the same 6 patients previously noted as having single-voxel level discrepancies (Fig. 3B). All 6 positive outliers had an Agatston score of < 5 by expert calculation, where the AI found zero. This resulted in a 25–78% difference in MESA percentile despite the calcium discrepancy being so small.

## Discussion

The purpose of this study was to comprehensively evaluate the performance of a deep learning algorithm for CACS with a focus on accuracy, efficiency, and downstream clinical reliability. The most important finding is repeat validation of accuracy of the AI algorithm for Agatston scores, the expert and AI demonstrating inter-expert level reliability. Unbalanced accuracy for qualitative calcium score severity was greater than 90%, including a 100% accuracy for CACS = 0. The AI algorithm also was found to save time compared to manual segmentation in every case, including in cases where CAS = 0. The magnitude of this difference increased with calcium burden, as AI time was independent of calcium burden. Finally, the AI derived scores were clinically robust, with 95% of AI-derived MESA percentiles being within 10% of the true value.

The first question asked regarding the utility of any image interpretation tool is accuracy. AI CACS have been exceptional in this space, with recent reviews identifying ICCs ranging between 0.84 and 0.99 [15]. This work builds off of previous assessments which reported excellent global and vessel-specific agreement (ICC = 0.904; 95% CI 0.857–0.936), a finding which is again validated in this study [10, 12]. While the interpretation of ICC in radiology can be contentious and is by definition arbitrary, ICCs of > 0.9 are generally considered “Excellent” and approximate expert level [16]. Indeed, inter-expert reliability in some cases was noted to be 0.85 or below [15]. The variability observed between AI methods and between AI and expert assessments can be attributed in part to the inherent limitations of the Agatston score itself, which is influenced by factors such as image resolution, slice thickness, and calcium density. Additionally, this study demonstrated qualitative assessment of calcium scoring was excellent by weighted agreement and absolute severity classification (mild, moderate, etc.). Indeed, this qualitative assessment of severity fluctuates greatly in the literature between k = 0.5 and k = 0.91 depending on the specific parameters [15]. Taken together, the authors argue that for the express purpose of calcium scoring on dedicated ECG-gated CT scans with these acquisition parameters, this AI algorithm performs close to the expert level. More study is needed to apply these findings outside of routine ECG-gated calcium scoring CT.

AI demonstrated high agreement with expert radiologists (ICC = 0.951; 95% CI 0.933–0.964), which is comparable or higher to the reported inter-observer variability among radiologists, typically ranging from 0.85 to 0.95. Radiologist variability often increases in challenging cases with higher CACS, while the AI maintained consistent performance across all calcium levels. Human factors such as fatigue and experience can lead to variability, but the AI’s standardized approach helps reduce these inconsistencies, particularly in routine cases. This consistency, especially in cases with minimal or significant calcification, suggests the AI could improve reliability and efficiency in high-volume clinical settings.

Understanding that accuracy is preserved, the next question becomes does a tool improve efficiency. Calcium scoring can be quite tedious, especially in the case of large-volume, multifocal coronary calcium with concomitant distractors such as mitral annulus or pericardial calcifications [17]. Regardless, cardiac imagers who have interpreted these exams know firsthand that these exams may take more time relative to complexity than most realize. The AI literature reflects this finding with heterogenous computational times ranging from 2 s to 10 min [15]. In this study the algorithm reports a consistent median time of 15 s for total calculation beginning from initiation of the module to the end of the standardized reporting of results. For reference, the fastest model in the literature reports a time of ~ 1.5 s and compares well with other non-cloud based [15, 18]. Importantly, the time to interpretation for the AI was always faster than the expert calculations and did not vary based off of calcium burdens, whereas the expert segmentation time increased with total Agatston score. Among all other findings presented, this may be the most advantageous result.

An important point is that the cardiac imagers are ultimately responsible for the accuracy of any included CACS in the final report, necessitating a semiautomatic evaluation for comparison as well. This was performed by first using the AI as in the automated step, but with manual review and adjustment as needed. Noted discrepancies were minor, often on the order of a single plaque area or even a single voxel. Nevertheless, time to interpretation increased with this group with only marginal benefit. Manual time to interpret was still higher for those with Agatston scores > 10, suggesting that semiautomatic review is still beneficial for many patients. Indeed, the authors suggest this approach currently. One way to maximize this approach is to have the CT technologist initialize the AI module at the workstation or have the post-processing automatically initiate, thus streamlining review and revision as needed.

Lastly, CACS is part of other risk stratification systems such as the MESA score. Reliable clinical translation is therefore one of the most important features of a durable AI application. In this study the authors found excellent correlation between the expert and AI-derived MESA scores with a mean bias of 2.1%. Furthermore, 95% of all calcium error resulted in less than a 10% discrepancy in MESA score, suggesting high fidelity clinical translation. Systematic analysis of error found that the largest errors came from minute (Agatston score < 5) changes in young patients where single-digit Agatston scores assigns them a high percentile. While these discrepancies were minor, they led to increased MESA score percentiles for some patients, highlighting the importance of further refinement in AI algorithms to improve sensitivity, particularly for minimal calcifications that may have clinical relevance. Caution and common sense should be exhibited in this group as these patients reflect the edge of scientific understanding about developing coronary artery disease [19, 20].

Limitations of this study primarily come from the narrow patient selection and acquisition. All patients were derived from routine dedicated calcium scoring exams, and thus were all outpatient, reasonably healthy, and performed using standard acquisition protocols with ECG-gated examinations. Efforts were made using sequential inclusion to capture a “true clinical population,” but there is inherent selection bias in clinic referrals. Additionally, deviations in acquisition protocols have been demonstrated to be important in the literature, and these results should not necessarily be applied to non-gated examinations [21]. One additional consideration not evaluated is the transmission time for images from the picture archive and communication system to the post-processing software. This step represents an additional point of inefficiency that was not evaluated in this study as this step was required for both manual and automatic scoring and as such does not differ between the methods. An intermediate step from the CT scanner to automatically initiate the post-processing software may be a further method to promote efficiency and is beyond the scope of this study. An additional limitation is the comparison to the MESA study, which was conducted on older, lower-resolution scanners. While MESA remains a standard for cardiovascular risk assessment, differences in scanner technology may influence CACS, and these discrepancies have been shown to affect patient risk categorization in several studies. Lastly, this study was powered for global assessment of calcium score, and thus vessel specific measurements were omitted. Ongoing research in this area both in clinical and technological development may necessitate further refinement [22].

In conclusion, while the AI algorithm demonstrated excellent performance in calcium scoring, offering significant time savings and consistency across all calcium burden levels. Minor discrepancies should be considered in specific clinical contexts, particularly in younger or low-risk patients. Overall, the AI tool holds promise for routine clinical use, reducing variability and improving efficiency in high-volume settings. However, a semi-automatic approach remains beneficial for ensuring optimal accuracy, especially in cases where minimal calcifications may impact clinical decision-making.

## Data Availability

No datasets were generated or analysed during the current study.

## Abbreviations

AI: Artificial Intelligence
CACS: Coronary Artery Calcium Scoring
CT: Computed Tomography
CAD: Coronary Artery Disease
ECG: Electrocardiogram
ICC: Intraclass Correlation Coefficient
MESA: Multiethnic Study of Atherosclerosis
MDCT: Multi-Detector Computed Tomography
ROI: Region of Interest
HU: Hounsfield Units
IQR: Interquartile Range
